# Diagnostic experiences of Duchenne families and their preferences for newborn screening: A mixed‐methods study

**DOI:** 10.1002/ajmg.c.31992

**Published:** 2022-08-09

**Authors:** Norah L. Crossnohere, Niki Armstrong, Ryan Fischer, John F. P. Bridges

**Affiliations:** ^1^ Department of Biomedical Informatics The Ohio State University College of Medicine Columbus Ohio USA; ^2^ Parent Project Muscular Dystrophy Washington District of Columbia USA; ^3^ Present address: Department of Internal Medicine Division of General Internal Medicine, The Ohio State University College of Medicine Columbus Ohio USA

**Keywords:** diagnostic odyssey, Duchenne muscular dystrophy, newborn screening, preferences

## Abstract

Duchenne muscular dystrophy is the most common form of muscular dystrophy diagnosed in childhood but is not routinely screened for prenatally or at birth in the United States. We sought to characterize the diagnostic experiences of families and describe their preferences for newborn screening (NBS). We conducted a registry‐based survey of families with Duchenne and Becker muscular dystrophy that included open‐ and closed‐ended questions regarding the journey to a diagnosis, preferences for when to learn of a diagnosis, and how knowledge of a diagnosis would impact life decisions. Open‐ended responses were analyzed thematically, and closed‐ended responses were analyzed descriptively. Sixty‐five families completed the survey. The average ages of first concern and diagnosis were 2 and 4 years, respectively. One‐third of families (30%) indicated that they would prefer to receive a diagnosis in the newborn period irrespective of treatment options available, and nearly all of the remaining families (93%) indicated that they would want to learn about a diagnosis if there were treatments that worked well during the newborn period. All families (100%) indicated that a diagnosis in the newborn period would impact life decisions. We identified three overarching themes, which described the stages of the diagnostic journey, including having concerns about the child, seeking answers, and receiving the diagnosis. NBS can facilitate improved health outcomes through early access to care, and inform families on major health and nonhealth decisions. The preferences and experiences of families and other stakeholders should be considered when determining the potential value and benefit of expanding NBS programs.

## 1 INTRODUCTION

Duchenne muscular dystrophy (Duchenne) is a rare, genetic neuromuscular disorder for which there is currently no cure. Duchenne is the most common form of pediatric muscular dystrophy, occurring in 1 in 5000 live male births each year (Mah et al., 2014). Duchenne is characterized by muscle weakness that leads to loss of ambulation (typically in the teen years) and eventually to pulmonary and cardiac complications, which are often the causes of death (Stromberg, Darin, Kroksmark, & Tulinius, 2012). The condition is caused by a pathogenic variant in the X‐linked dystrophin gene, which is vital for protein development and muscle function (Hoffman, Brown, & Kunkel, 1987). Two‐thirds of people with Duchenne inherit the pathogenic variant, while the remaining third develop the disease from a de novo pathogenic variant (Lee et al., 2014).

Newborn screening (NBS) is a public health service that tests for rare genetic, hormone‐related, and metabolic disorders using a blood test soon after birth. The goal of newborn screening is to identify, as early as possible, fatal or disabling conditions (What is the purpose of newborn screening? Eunice Kennedy Shriver National Institute of Child Health and Human Development, 2022). Doing so can promote early access to treatments to reduce or eliminate the effect of a condition and improve quality of life. The Recommended Uniform Screening Panel (RUSP) provides guidance to US states on which conditions to screen for as a part of their NBS programs. Conditions are selected for inclusion on the RUSP based on the net benefit of screening, the certainty of the evidence, and the feasibility/readiness of expanding NBS programs to include the condition (Kemper et al., 2014; Committee Approach to Evaluating the Condition Review Report (Decision Matrix). US Health Resources & Services Administration, 2022). There are currently 35 core conditions included on the RUSP, the most recent addition being spinal muscular atrophy in 2018. In February 2022, the RUSP voted to include MPS II (Hunter Syndrome), and this change will be made upon the final approval of the secretary of Health and Human Services. Being included in the RUSP is an important step to improving the surveillance of a given rare condition.

Duchenne is not routinely screened for at birth or prenatally in the US. Several NBS pilots for Duchenne are underway or have recently been completed, (Parad, Sheldon, & Bhattacharjee, 2021; Wynn et al., 2022) and a nomination package to include Duchenne on the RUSP is in preparation. Screening tests are highly sensitive to detect variants of Duchenne for which there are current therapies (Beckers et al., 2021). In the absence of NBS, many patients with Duchenne (as well as those with Becker, a related but milder form of muscular dystrophy) experience diagnostic odysseys which have spillover effects on the family including causing psychosocial distress, financial burdens, and quality of life. Although signs of Duchenne may be visible as early as when boys begin walking, Duchenne diagnosis typically occurs around the age of four to five, and after a two‐year diagnostic delay (van Ruiten, Straub, Bushby, & Guglieri, 2014; Wong, McClaren, Archibald, et al., 2015). Variation in diagnostic age has been observed within (Holtzer et al., 2011) and across countries (D'Amico, Catteruccia, Baranello, et al., 2017), and by race/ethnicity (Counterman, Furlong, Wang, & Martin, 2020). Late diagnosis for Duchenne can delay adherence to standards of care, such as by delaying access to corticosteroids which are ideally started before the plateau phase at four to five years (Merlini et al., 2012; Messina & Vita, 2018). Additionally, novel exon‐skipping therapies for Duchenne are approved for patients of all ages with qualifying pathogenic variations and are more effective at increasing dystrophin production when significant muscle tissue is still present (Kharraz, Guerra, Pessina, Serrano, & Muñoz‐Cánoves, 2014). Too often, patients with Duchenne are not diagnosed until they have already lost substantial muscle tissue (Vill et al., 2020).

In this study, we sought to characterize the experiences of families with regard to receiving a diagnosis of Duchenne, and their perspectives on how NBS and diagnosis in the newborn period would affect a range of decisions. Prior research of family attitudes towards NBS in Duchenne have indicated that families have historically endorsed the use of NBS, even before treatments for Duchenne were available (Firth & Wilkinson, 1983). More generally, research has observed mixed attitudes towards NBS (Conway, Vuong, Hart, Rohrwasser, & Eilbeck, 2022). Providing a more detailed understanding of Duchenne diagnostic experiences can inform decision‐makers concerning the value of NBS for Duchenne.

## 2 METHODS

We conducted a registry‐based survey of families with children with Duchenne or Becker regarding their experience with receiving a diagnosis and their perspectives on the value of NBS. The survey was web‐based, and sent to registered families in the Duchenne Registry who were (a) a parent of a living child (of any age) with a reported diagnosis of either Duchenne or Becker, and (b) residing in the United States. The recruitment email was sent to a total of 2059 email addresses (each representing a unique patient record) on two occasions. Individuals who initiated the survey but did not complete it were sent up to two reminders to finish their responses.

The Duchenne Registry is a patient self‐report registry for individuals with Duchenne or Becker and carrier females (Rangel, Martin, & Peay, 2012). Registry participants (patients 18 years and older or parents/custodians/legal guardians of children under the age of 18) complete survey questionnaires on health‐related topics including diagnosis, mobility and musculoskeletal function, respiratory and cardiac function, and therapies. Data are maintained in a HIPAA‐compliant database. All participants have granted permission for de‐identified information shared in the registry to be provided to researchers.

The survey sent to registry participants contained both open and closed‐ended questions (see Supplemental Material for survey questionnaire). Open‐ended questions asked families to describe (a) their journey towards receiving a diagnosis, as well as (b) how having received a diagnosis in the newborn period would have been helpful, and (c) difficult for their family. Closed‐ended questions assessed at what age families would have liked learning about their child's diagnosis. If families did not prefer to learn of their child's diagnosis as a newborn, they were probed whether they would want a diagnosis as a newborn if there was a treatment available for use in the newborn period. Families also indicated what life decisions would have been affected by learning of their child's diagnosis as a newborn. Families were asked to provide the email address linked to their child's Duchenne Registry account so that researchers could link their responses with information from their Duchenne Registry records. Families indicated their consent to participate on the first screen of the survey. No compensation was provided.

We analyzed responses to open‐ended questions thematically. We first read all responses, familiarizing ourselves with the data. We then reviewed the responses paying attention to emerging themes. Any potential themes were listed and then cleaned and condensed. Direct quotes from families were selected to represent each theme. We analyzed the responses to the open‐ended questions collectively as, upon reviewing them, we observed that they reflected similar themes. We analyzed closed‐ended questions descriptively. This included descriptively comparing how life decisions perceived to be affected by newborn diagnosis varied across families who preferred and did not prefer a newborn diagnosis. We did not use statistical tests for these across‐group comparisons as we did not have a priori hypotheses about how the groups would differ. All study procedures were approved by the Geisinger Institutional Review Board, including those specifically for The Duchenne Registry and its electronic consent (IRB#2014–0621) and those specific to the current survey (IRB#2021–0943).

## 3 RESULTS

One‐hundred and sixty‐five individuals opened the survey and 110 completed screening questions to participate. Sixty‐five families completed the first question of the survey describing their family's journey to a Duchenne diagnosis. These families formed the analytic sample. Sixty‐four completed the question asking how a newborn diagnosis would have been helpful, and sixty‐three completed the question asking how a newborn diagnosis would have been difficult. In closed‐ended questions, all respondents indicated their preference for the age to learn of diagnosis. All surveys were linked to a Duchenne Registry account.

### 3.1 Quantitative findings

**FIGURE 1:**
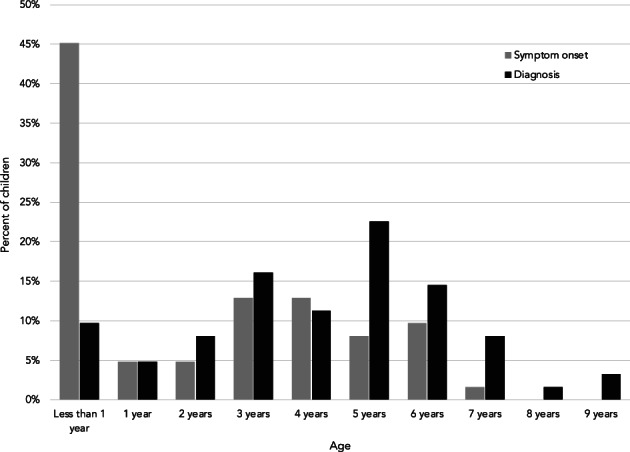
Age of symptom onset and diagnosis across the sample

**FIGURE 2:**
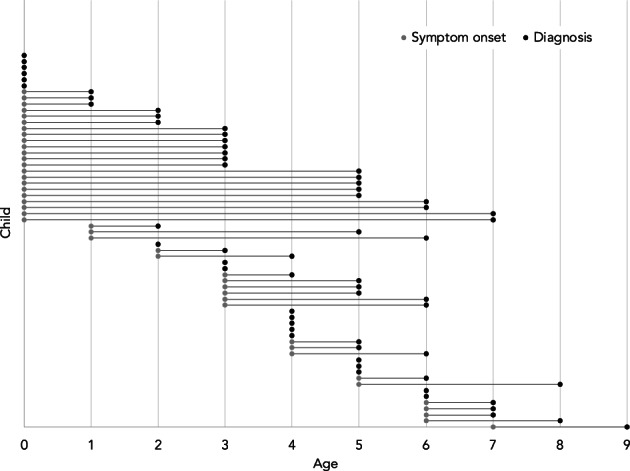
Age of symptom onset and diagnosis for each child. Each bar represents the length of the diagnostic journey for one child. For instance, if symptom onset begins at age one (represented by a gray dot), and diagnosis is at 6 years (represented by a black dot), the length of the diagnostic journey for that child was 5 years.

Most families reported their child's diagnosis to be Duchenne (84%) or Becker (8%; Table 1) muscular dystrophy. For nearly a tenth of patients, it was still unclear if their diagnosis was Duchenne or if it was Becker (8%). The mean age of diagnosis was 4.0 years (ranging from before birth to 9 years old). The mean onset of symptoms/concerns was 2.1 years (ranging from before birth to 7 years), indicating that the average diagnostic journey lasted 1.9 years (range 0–7 years). Figure 1 visualizes the age of symptom onset and diagnosis across the entire sample, and Figure 2 visualizes the length of the diagnostic journey (time from symptom onset to diagnosis) for each child. Difficulty with physical activities was the most common initial concern (62%), along with delays in motor development (48%), enlarged calves (38%), and frequent falls (35%). An eighth of families (14%) indicated that concerns about the child's health were raised by their school/preschool/daycare before the diagnosis.

**TABLE 1:** Characteristics of child

	Total *n* = 65	Do not prefer newborn diagnosis *n* = 45	Prefer newborn diagnosis *n* = 20
Diagnosis, *n* (%)			
Duchenne	55 (85)	39 (87)	16 (80)
Becker	5 (8)	4 (9)	1 (5)
Unclear if Becker or Duchenne	5 (8)	2 (4)	3 (15)
Age of diagnosis, mean (range)	4.0 (0–9) years	4.3 (0–9) years	3.4 (0–7) years
Age of first concerns, mean (range)^a^	2.1 (0–7) years	2.4 (0–7) years	1.7 (0–5) years
Length of diagnostic odyssey, mean (range)^a^	1.9 (0–7) years	2.1 (0–7) years	1.8 (0–7) years
Physical concerns first noticed, (all that apply), *n* (%)			
Difficulty with physical activities	40 (62)	28 (62)	12 (60)
Delays in motor development	31 (48)	21 (47)	10 (50)
Enlarged calves	25 (38)	19 (42)	6 (30)
Frequent falls	23 (35)	18 (40)	5 (25)
Toe walking	16 (25)	13 (29)	3 (15)
Delays in developing language	16 (25)	11 (24)	5 (25)
Elevated CK level	13 (20)	10 (22)	3 (15)
Elevated liver enzymes	9 (14)	7 (16)	2 (10)
Muscle pain/cramps	8 (12)	7 (16)	1 (5)
What prompted concerns, (all that apply), *n* (%)			
School/preschool/daycare raised concern	9 (14)	5 (11)	4 (20)
Family member with muscular dystrophy	8 (12)	3 (7)	5 (25)
Newborn/infant screening	2 (3)	0 (0)	2 (10)
Health care provider raised concern	4 (6)	4 (9)	0 (0)

Abbreviation: CK, creatine kinase.

^a^ Missing for *n* = 3, all of whom did not prefer newborn diagnosis.

One‐third of families (30%) would have preferred to learn of their child's diagnosis before the child was 6 months old (i.e., learn when the child was a “newborn”). Of the remaining families, 17% preferred to learn when the child was a baby/toddler (6–24 months), 9% preferred to learn between 2 and 4 years, 12% preferred to learn between 4 and 6 years, and 33% indicated that they did not know what age they would have preferred to learn of the diagnosis. Among those who did not indicate that they preferred to learn when the child was a newborn, 93% indicated that if there were a treatment that worked best when the child was a newborn, they would want their child diagnosed in the newborn period. Families who preferred not to receive a diagnosis in the newborn period had children who were diagnosed later on average than those who preferred to receive their child's diagnosis in the newborn period (4.3 vs. 3.4 years, Table 1).

**FIGURE 3:**
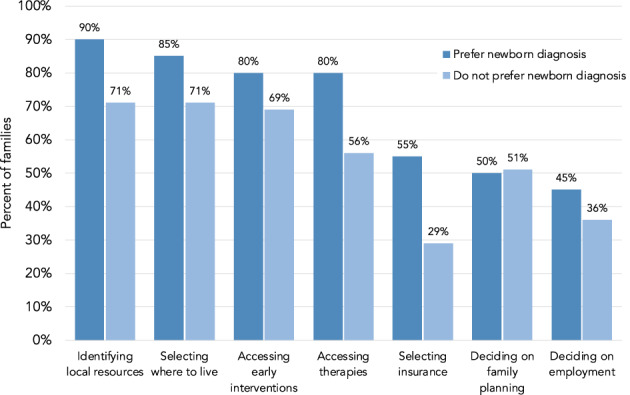
Decisions that would be influenced by receiving a diagnosis in the newborn period

All families indicated that learning of the diagnosis as a newborn would have informed at least one major decision. The most highly endorsed decision that would have been affected by a newborn diagnosis was deciding to seek out support and community resources (76%), followed by making decisions about where to live (74%), considering clinical trial participation (71%), getting access to approved therapies sooner (62%), and making decisions about family planning (50%), employment (38%) and health insurance (36%). Those who did not prefer diagnosis as a newborn were less likely to indicate that the diagnosis would have affected any given decision as compared to those who preferred diagnosis as a newborn (Figure 3). The exception to this was family planning; both groups were evenly split on whether or not receiving the diagnosis as a newborn would have influenced the decision to have more children.

### 3.2 Qualitative findings

We identified three overarching themes which described the stages of the diagnostic journey, including having concerns about the child, seeking answers, and receiving the diagnosis. These themes, their subordinate themes, and example quotes are explored below and summarized in Table 2.

**TABLE 2:** Themes in a families' journeys towards a muscular dystrophy diagnosis

Themes	Illustrative quote
Having concerns	
Family history	“When my son was born I asked since his brother had Duchenne as well. The pediatrician said just enjoy my baby and if things become apparent to then do one. At age three I felt he wasn't doing the things he should and he was having difficulty keeping up with his cousins.”–Mother of son with Duchenne, diagnosis at 3
Outside observers	“His preschool teacher mentioned that he was slower than his peers to get up from the floor and running around on the playground.”–Mother of son with Duchenne, diagnosis at 5
Wait‐and‐see	“Although his pediatrician was wonderful, she never screened for development, or was concerned ‘he's just not a gross motor kid.’”–Mother of son with Duchenne, diagnosis at 3
Seeking answers	
Feeling dismissed	“As a mom, I just knew something wasn't right. I pushed the pediatrician to reconsider, to run more tests, to just figure it out. By this time, [child] was falling behind. He could not keep up with his cousins and he struggled to get up off of the floor.”–Mother of son with Duchenne, diagnosis at 5
Numerous evaluations	“My son was diagnosed when he was a little over 5 years old after we had consulted five doctors to get a diagnosis.”–Mother of son with Duchenne, diagnosis at 5
Health system delays	“Initially they asked if we had a neurologist we wanted to see…the wait for a local one was 3 months.”–Mother of son with Duchenne, diagnosis at 5
Receiving diagnosis	
Incidental/accidental diagnosis	“Genetic counselors were brought into his case to try to determine if his brain damage had any genetic cause. It did not, but they found a muscular dystrophy diagnosis in the process.”–Mother of son with Becker, diagnosis in first year of life
Provider communication	“The neurologist splayed her hands helplessly before her and said, ‘these things have to start in families somewhere.' It felt very callous, but after the fact, I wondered how else she might have told me.”–Mother of son with Duchenne, diagnosis at 7
Sources of health information	“The neurologist gave us the final diagnosis over the phone. He provided a lot of information and said to stay off of Google for our research.”–Mother of son with Duchenne, diagnosis at 4

### 3.3 Having concerns

There were several reasons why families initially became concerned for their child's health. Some families expressed having a family history of muscular dystrophy. One parent indicated that their partner had undergone preconception screening given a family history of Duchenne, but the test only screened for a specific pathogenic variant that the child did not have. In the words of the father, *“unfortunately they did the testing only for exon deletion…we were not aware of any such thing like exon…duplication.”* The family subsequently did not learn of their son's muscular dystrophy diagnosis until age 4, at which point they had difficulty enrolling their son into clinical trials.

For other families, outside observers provided the first indication that the child may not be developing as would typically be expected. Families recalled how other members of the family, such as grandparents, raised concerns. Several families described specifically how educators, and specifically those from daycare and preschool, were the first to bring the concern to their attention. A father whose child was cared for by a one‐on‐one nanny rather than by a daycare reflected that *“sometimes I wonder if a daycare teacher might have noticed something wrong with him sooner than we eventually did. It was when he finally started preschool that the principal told us that he seemed to struggle to climb up the stairs.”* His son was not diagnosed with Duchenne until age 4.

Some families described how concerns with their child's functioning were initially attributed to general and unspecified delays in meeting developmental milestones. This was sometimes attributed to a general motor delay and families were told to “wait‐and‐see,” which unfortunately delayed their care. A mother whose son received a diagnosis of Duchenne at age 3 recalled that while the child was perceptively behind on milestones by as early as 6 months and continuing through nearly 2 years, the *“pediatrician just kept telling me that he was just slightly behind and he was just developing at his own pace.”* Other families indicated that their child hit milestones in a relatively timely matter, which further delayed diagnosis. “*Some of his milestones were slightly delayed, but he always got there before anyone started to really worry… [the doctor] told us to come back if he still wasn*'*t walking by 18 months. He took his first independent steps at exactly 18 months old…. I think we just missed getting his diagnosis back then,”* as described by a mother whose son was diagnosed with Duchenne at age 5.

### 3.4 Seeking answers

Many families faced challenges in finding answers even once they recognized that there may be something amiss with their child's health. Families expressed feeling dismissed by clinicians. One mother whose son was diagnosed at with Duchenne at age 7 said: *“I was frustrated at being dismissed. I knew something was wrong but no one would take me seriously. This went on for years.”* Another mother, whose son was diagnosed with Duchenne at age 5, described that a doctor invalidated her concerns, making her feel like she and her husband were *“expecting too much from our son.”*

Many families sought multiple consultations before receiving a diagnosis. The search for answers sometimes caused tension at home. One mother whose son received his Duchenne diagnosis at age 3 described these appointments and her family's perspective on her search for answers: “*I went to many pediatricians, no one listened. I went to [an] orthopedic specialist who assured me there was nothing wrong. I saw [physical therapies] and [occupational therapists] everyone said there is nothing wrong just a little gross motor delay. My family asked me to stop. They said I was inviting bad luck*.” Another mother whose son was diagnosed with Duchenne at age 7 expressed that searching for the cause of the developmental delays caused her family *“constant concern.”*

Families also experienced delays in diagnosis as a result of health system factors, such as difficulties in working with insurance companies to reimburse genetic testing, long processing times for genetic testing results, and limited availability of neurologists and other specialist clinicians. Families described waiting months to be seen by a clinician, only to be referred elsewhere with another months‐long waiting list. Upon begin appropriately referred, the diagnosis was often swift. One family who was eventually referred to a specialty gait clinic offered a detailed description of their experience:

“*We showed up for our appointment… prepared for a multi‐hour assessment. The room had clear panels on the floor, with cameras underneath. They had all kinds of ways to assess gait through various walking, running, etc.…after not even 5 minutes, the orthopedic surgeon in charge shut the entire thing down and asked us to meet him in an exam room…he said that [my child] has muscular dystrophy*.”

This mother, whose son was 9 years old when he received his diagnosis of Becker muscular dystrophy, went on to describe that her son was immediately connected to physical therapy and other services.

### 3.5 Receiving the diagnosis

The communication of the diagnosis itself was a watershed moment for many families. Families stated that there was no good way to learn that their child had muscular dystrophy, while also acknowledging that certain aspects of provider communication improved or worsened the delivery of the news. A mother whose son received his Duchenne diagnosis at age 7 described how, “*The neurologist splayed her hands helplessly before her and said*, “*These things have to start in families somewhere*.” *It felt very callous, but after the fact*, *I wondered how else she might have told me*.” A mother of a child diagnosed with Duchenne when he was 5 years old described that *“the most heart‐wrenching moment of our lives…was put on display”* for the numerous medical students and trainees in the room. Several families reported being told of their diagnosis through a genetic counselor, who received generally favorable evaluations. In the words of a mother whose son with Becker was diagnosed when he was 4, *“My genetics counselor was instrumental in learning more about what this variant might mean for our family.”*

Families described that their doctors offered mixed perspectives on searching for muscular dystrophy on the internet. One mother whose 3‐year‐old son was diagnosed with Duchenne recalled a provider describing internet research as a given: *“‘Look,” the doctor said, “I know you*'*re going to look it up on the internet, so I*'*ll just tell you. Boys with Duchenne are wheelchair‐bound by 12 and die in their mid‐twenties.’”* Others indicated that the providers advised them to stay off the internet when researching the condition. A mother whose son was 3 at the time of his Duchenne diagnosis told her husband to avoid looking for information online, and that *“the internet is a scary place.”*

Several families learned of their child's diagnosis as an incidental finding of another medical test. A mother of a son with Becker diagnosed in his first year of life described that the muscular dystrophy diagnosis was made by genetic counselors who were searching for a cause to unrelated brain damage. Others reported that they learned of the diagnosis through what they had been told was routine prenatal carrier screening. A mother who received notice of her child's muscular dystrophy diagnosis before his birth reported that, *“we are very lucky that he was diagnosed before birth, which allowed us to get him treatment as soon as possible.”*

## 4 DISCUSSION

Nearly all families of in this study expressed that they would like to learn of a muscular dystrophy diagnosis in the newborn period if there were treatments that were effective at this age. All families indicated that learning of their child's diagnosis early in life would have helped them to make major life decisions. The length of the diagnostic journey and ages of onset and diagnosis reported by families in the current study are consistent with previous research (van Ruiten et al., 2014; Wong et al., 2015). Additionally, the themes identified from qualitative analysis including prolonged diagnostic odyssey, feeling dismissed by medical communities, and sensitivities around the communication of the diagnosis itself, are consistent with prior research on the experiences of parents of children with Duchenne (Bendixen & Houtrow, 2017; Bendixen, Morgenroth, & Clinard, 2016).

It is notable that in the current study only 30% of families were initially interested in receiving a diagnosis in the newborn period, but nearly all were in favor of receiving an early diagnosis if it meant earlier access to treatments that worked well at the newborn stage. Therapies for Duchenne are typically most effective before significant muscle deterioration. Gene therapies are routinely administered in infancy for spinal muscular atrophy, (Hoy, 2019) and there is emerging data to suggest that administration in infancy might be similarly effective in muscular dystrophy. Early intervention services such as physical, occupational and speech therapy all yield improved outcomes when started at earlier ages. Collectively, this suggests that more efforts are needed to convey to famililes that even now, before curative treatments are available for muscular dystrophy, newborn diagnosis can facilitate improved long‐term health outcomes.

The time is ripe to consider how the preferences of families and other stakeholders should be used to inform NBS programs. Over the past decade, there has been tremendous growth in the scientific study of preferences to inform all stages of medical decision‐making, from the development and evaluation of novel therapies to the delivery of clinical care and the implementation of health policy (Whichello et al., 2020). Preference information is systematically incorporated into health policy decision‐making at the regulatory level by groups such as the US Food and Drug Administration (FDA) (CDRH F, 2016). Notably, guidance from FDA outlines that the preferences of diverse individuals including families, professionals and the public ought to be elicited to inform decision‐making. Calls have been made to modernize the process of NBS and how considtions are selected to be on the RUSP in order to facilitate timely access to treatments once they become available (Andrews, Porter, Bailey, & Peay, 2022; Bailey et al., 2021; Ciafaloni et al., 2009; Thomas, Conway, Fapo, et al., 2022). An approach to modernizing the RUSP might include an updated integration and assessment of preferences for NBS. Research has explored preferences for NBS, (Lipstein et al., 2010; Miller et al., 2015; Tarini et al., 2018; Vass, Georgsson, Ulph, & Payne, 2019; Wright, Ulph, Dharni, & Payne, 2017; Wright, Ulph, Lavender, Dharni, & Payne, 2018) however preference assessment is not routinely incorporated into decisions regarding which conditions to include on the RUSP. Evidence gaps exist regarding the role of preferences and NBS programs, including uncertainty regarding how preference information can be integrated into the evidence review process for the RUSP, and how families, professionals, and the public may differently value preference information in the NBS context.

The current study used a direct‐elicitation approach to explore preferences for NBS in muscular dystrophy. Numerous approaches outside of direct‐elicitation can be used to study preferences in health, including but not limited to quantitative methods such as discrete‐choice experiments and best‐worst scaling, as well as qualitative methods (Medical Device Innovation Consortium (MDIC), n.d.). The Duchenne community has been a leader in measuring preferences, (Bridges et al., 2019; Crossnohere, Fischer, Crossley, Vroom, & Bridges, 2020; Crossnohere, Fischer, Lloyd, Prosser, & Bridges, 2021; Hollin, Peay, Apkon, & Bridges, 2017; Hollin, Peay, Fischer, Janssen, & Bridges, 2018; Peay, Hollin, & Bridges, 2016; Peay, Hollin, Fischer, & Bridges, 2014; Schuster, Crossnohere, Fischer, Furlong, & Bridges, 2022) including those specific to inform therapeutic benefit–risk decisions (Bridges et al., 2018; Crossnohere, Fischer, Vroom, Furlong, & Bridges, 2022). Future research should apply these methods to study preferences for NBS.

Families' experiences with and preferences for diagnosis can play an important role in informing the development of new policies for NBS. We examined the attitudes of families of children who stood to receive a diagnosis from the expanded NBS panel, but the reality is that all families with newborn children–not just those with a detected condition–stand to be impacted by the decision to expand the panel. A recent study of preferences for NBS among parents of healthy babies (Armstrong et al., 2022) indicated that over 80% of parents want to be told of conditions detected by NBS if they could be treated. Only half indicated they would want to know if the condition could not be treated. The general public's preferences for expanded NBS is essential to promote overall programmatic success. Engaging the public is particularly essential in the light of increased interest in transitioning NBS to use genomic sequencing approaches, which have the potential to screen for thousands of additional disorders.

This study was limited by its retrospective nature which asked families to report on their diagnostic journey after having gone through the process. Results are subject to recall bias, as families may have perceived an exaggerated relationship between risk factors and the muscular dystrophy diagnosis. Additionally, this study posed questions that were hypothetical in nature, such as asking families to consider what life decisions might have been impacted had they received a diagnosis as a newborn. Results from these questions are subject to hypothetical bias and may not reflect the actual decisions that families would have made had they learned about their child's diagnosis at the newborn stage.

## 5 CONCLUSION

There continues to be a delay in the diagnosis of Duchenne muscular dystrophy which profoundly affects families and reduces their opportunities to access early interventions. Diagnosis of Duchenne during the newborn period would inform families in making major life decisions. Integrating the perspectives and preferences of families to inform NBS policy and decisions about which conditions are included in the RUSP would modernize the system to be consistent with public health policies of other national organizations such as the FDA.

## CONFLICT OF INTERESTS

RF and NA are employees of Parent Project Muscular Dystrophy. NLC and JFPB have received funding from PPMD.

## Supporting information


**Appendix S1** Supporting Information.

## Data Availability

The data that support the findings of this study are available on request from the corresponding author. The data are not publicly available due to privacy or ethical restrictions.
