# Supplementary material for: Diagnostic experiences of Duchenne families and their preferences for newborn screening: A mixed‐methods study
Source: Am J Med Genet C Semin Med Genet. 2022 Aug 9;190(2):169–77. doi: 10.1002/ajmg.c.31992 (PMC9804254; doi:10.1002/ajmg.c.31992)
Supplement: Supplementary file 1 — Appendix S1 Supporting Information. [file AJMG-190-169-s001.docx]

Supplemental materials for:

**Diagnostic experiences of Duchenne families and their preferences for newborn screening: A mixed-methods study** In *American Journal of Medical Genetics Part C*

Norah L Crossnohere^1,2§^

Niki Armstrong^3^

Ryan Fischer^3^

John F P Bridges^1^

^1^Department of Biomedical Informatics, The Ohio State University College of Medicine, Columbus, OH

^2^Department of Internal Medicine, Division of General Internal Medicine, The Ohio State University College of Medicine, Columbus, OH

^3^Parent Project Muscular Dystrophy, Washington DC

^§^Corresponding author:

Norah L Crossnohere, PhD

[Norah.crossnohere@osumc.edu](mailto:Norah.crossnohere@osumc.edu)

**Contents:**

Appendix A: Web-based survey

**Appendix A. Web-based survey**

Please include your email address tied to your child’s Duchenne Registry account so we can utilize your responses from the diagnosis questions

(free text response)

Your personal information will not be shared with anyone and we will not identify you by name or other specific personal information.

1. The following question is open-ended—please feel free to use as much or as little space as you need to write your response. Please tell us about your journey to your child’s confirmed diagnosis of Duchenne or Becker. Please feel free to include why and when you first became concerned about your child, who you first expressed those concerns to, what specialists and providers you saw along the way, how the diagnosis was finally made, and how you were told about the diagnosis. (free text response)
2. In thinking about your experiences, when would you have preferred to learn that your child has Duchenne?
   - In the newborn period (<6 months) (branch to 2.1)
   - As a baby or toddler (6-24 months) (branch to 2.2)
   - Ages 2-4 (branch to 2.2)
   - Ages 4-6 (branch to 2.2)
   - I don’t know (branch to 3)

**2.1** Why? (branch to 4) (can skip) (free text response)

**2.2** Why? (branch to 3) (can skip) (free text response)

1. What if we had a treatment that worked best if started or given in infancy? Would that make you more interested in learning about the diagnosis earlier? (branch to 4)
   - Yes, if we had a treatment that worked best in babies, then I would want my child diagnosed as a baby.
   - No, even if a treatment were shown to work best in babies, I would prefer to wait until my child was older before he was diagnosed.
   - I don’t know / I’m uncertain
2. Imagine learning the diagnosis when your child was a baby. How would that have been helpful to you or your family? (branch to 5) (can skip) (free text response)
3. Imagine learning the diagnosis when your child was a baby. How would that have been difficult for you or your family? (branch to 6) (can skip) (free text response)
4. If your child’s diagnosis had occurred in the newborn period, how might that have affected your life choices and decisions on any or all of the following? (choose as many as applicable)

- Enrolling in early Intervention services such as physical or speech therapy
- Seeking out support and community resources
- Making decisions about health Insurance
- Making decisions about where we live (type of home, proximity to extended family, proximity to healthcare providers, etc)
- Making decisions about employment
- Making decisions about family planning
- Considering clinical trial participation
- Getting access to approved therapies earlier
- Other (free text)
- I don’t know
- No changes in any of our life choices

1. Please check this box if you are interested in sharing your story in the future, or if you would be interested in participating in future efforts for Duchenne newborn screening.
